# Clinical information prompt-driven retinal fundus image for brain health evaluation

**DOI:** 10.1186/s40779-025-00630-2

**Published:** 2025-08-06

**Authors:** Nuo Tong, Ying Hui, Shui-Ping Gou, Ling-Xi Chen, Xiang-Hong Wang, Shuo-Hua Chen, Jing Li, Xiao-Shuai Li, Yun-Tao Wu, Shou-Ling Wu, Zhen-Chang Wang, Jing Sun, Han Lv

**Affiliations:** 1 Key Laboratory of Intelligent Perception and Image Understanding of the Ministry of Education, School of Artificial Intelligence, Xidian University, Xi’an, 710071 China; 2 Guangzhou Institute of Technology, Xidian University, Guangzhou, 510555 China; 3 Department of Radiology, Kailuan General Hospital, Tangshan, 063000 Hebei China; 4 Shenzhen Bay Laboratory, Shenzhen, 518132 Guangdong China; 5 Department of Cardiology, Kailuan General Hospital, Tangshan, 063000 Hebei China; 6 Department of Radiology, Beijing Tsinghua Changgung Hospital, School of Clinical Medicine, Tsinghua University, Beijing, 102218 China; 7 Department of Radiology, Beijing Friendship Hospital, Capital Medical University, Beijing, 100050 China

**Keywords:** Retinal fundus image, Brain volume, Brain health, Magnetic resonance imaging, Deep learning, Eye and brain connection

## Abstract

**Background:**

Brain volume measurement serves as a critical approach for assessing brain health status. Considering the close biological connection between the eyes and brain, this study aims to investigate the feasibility of estimating brain volume through retinal fundus imaging integrated with clinical metadata, and to offer a cost-effective approach for assessing brain health.

**Methods:**

Based on clinical information, retinal fundus images, and neuroimaging data derived from a multicenter, population-based cohort study, the KaiLuan Study, we proposed a cross-modal correlation representation (CMCR) network to elucidate the intricate co-degenerative relationships between the eyes and brain for 755 subjects. Specifically, individual clinical information, which has been followed up for as long as 12 years, was encoded as a prompt to enhance the accuracy of brain volume estimation. Independent internal validation and external validation were performed to assess the robustness of the proposed model. Root mean square error (RMSE), peak signal-to-noise ratio (PSNR), and structural similarity index measure (SSIM) metrics were employed to quantitatively evaluate the quality of synthetic brain images derived from retinal imaging data.

**Results:**

The proposed framework yielded average RMSE, PSNR, and SSIM values of 98.23, 35.78 dB, and 0.64, respectively, which significantly outperformed 5 other methods: multi-channel Variational Autoencoder (mcVAE), Pixel-to-Pixel (Pixel2pixel), transformer-based U-Net (TransUNet), multi-scale transformer network (MT-Net), and residual vision transformer (ResViT). The two- (2D) and three-dimensional (3D) visualization results showed that the shape and texture of the synthetic brain images generated by the proposed method most closely resembled those of actual brain images. Thus, the CMCR framework accurately captured the latent structural correlations between the fundus and the brain. The average difference between predicted and actual brain volumes was 61.36 cm^3^, with a relative error of 4.54%. When all of the clinical information (including age and sex, daily habits, cardiovascular factors, metabolic factors, and inflammatory factors) was encoded, the difference was decreased to 53.89 cm^3^, with a relative error of 3.98%. Based on the synthesized brain MR images from retinal fundus images, the volumes of brain tissues could be estimated with high accuracy.

**Conclusions:**

This study provides an innovative, accurate, and cost-effective approach to characterize brain health status through readily accessible retinal fundus images.

*Trial registration No*. NCT05453877 (https://clinicaltrials.gov/).

**Supplementary Information:**

The online version contains supplementary material available at 10.1186/s40779-025-00630-2.

## Background

The human brain is the highest command center of the nervous system and regulates the functions of memory, language, and movement through a complex and multilevel neural network. The optimal integrity of brain structure, including an appropriate brain volume, is considered as a key aspect of brain health, while a prominent reduction in brain volume (brain atrophy) is an important indicator of brain degeneration [1–4].

Conventionally, brain tissue volumes are quantitatively measured based on brain magnetic resonance imaging (MRI) data. However, the high cost and time-consuming nature of MRI techniques have always been a great concern [5–8]. Conducting large-scale brain MRI collection within the general population is often impractical. Furthermore, in resource-poor or remote settings without MRI equipment and specific scenarios such as the aerospace field, MRI-based measurements are far from reach. This issue has long troubled the scientific community and will negatively impact medical equity and humanity’s endeavors in aerospace exploration. Thus, further investigation is warranted to explore a cost-effective alternative method for estimating brain volume to evaluate brain health status within the general population.

Retinal fundus images are widely utilized in ophthalmologic practice. In comparison to brain MRI examination, the acquisition of retinal fundus images is characterized by lower costs, fewer preparation requirements, and shorter time. Moreover, in contrast to a heavy MRI scanner, the equipment for retina image collection is lightweight and portable. The retina and brain are intricately interconnected in terms of embryonic development and physiological anatomy [9, 10]. Thus, the retina may serve as a distinctive window into the brain’s structures, functioning as a cost-effective tool for evaluating brain health.

Recent studies have explored the eye-brain connections through an image-based deep learning approach. Xie et al. [11] identified the degenerative changes in the geometrical parameters of retinal microvasculature in Alzheimer’s disease (AD) and mild cognitive impairment (MCI) individuals, thus offering valuable insights for the diagnosis and clinical decision-making of AD and MCI. Additionally, Zhao et al. [12] investigated the cross-organ connections between the eyes and brain at both phenotypic and genetic levels, and further observed that retinal imaging traits exhibited significant predictive power for neuropsychiatric and neurological disorders. However, a general limitation of these studies is their exclusive focus on extracting quantitative features and genetic phenotypes from the original images, resulting in the underutilization of retinal and brain imaging data, while the high-dimensional information embedded within the images remains insufficiently explored.

In this study, we aim to propose a novel cross-modal correlation representation (CMCR) framework to elucidate the intricate co-degenerative relationships between the eyes and brain using retinal fundus images and brain MRI data obtained from a large-scale population-based cohort study. Clinical metadata are employed as prompts to enhance the accuracy of brain volume estimation from retinal fundus images. We hypothesize that, based on biological connections between the eyes and brain, the volumes of brain tissues can be accurately estimated using retinal fundus images together with clinical information.

## Methods

### Data source

This study was embedded in the KaiLuan Study (KLS), an ongoing multicenter prospective cohort study conducted in Northern China [13]. More than 170,000 individuals aged 18–98 years have been included since the launch in 2006 [14]. Standardized clinical data collection, including demographic questionnaires, physical examinations, and laboratory tests, has been performed biennially across 11 local hospitals since baseline. From 2020 onwards, a subset of participants was voluntarily enrolled in the Multi-modality MEdical imaging sTudy bAsed on the KaiLuan Study (META-KLS) for the collection of brain MRI and retinal fundus images [15]. Between 2020 and 2022, these participants underwent baseline examinations including both brain MRI and retinal fundus imaging. Specifically, the age and sex distribution of the participants were similar to that of the overall KLS population [16]. The standardized protocol for clinical information collection, retinal/brain imaging, and automated processing pipeline has been previously published [15].

The inclusion criteria for this study included: 1) subjects who had completed both baseline brain MRI and retinal fundus scans between 2020 and 2022; 2) subjects who did not have a clinical diagnosis of cardiovascular diseases, neurological disorders, neurodegenerative diseases, or neuropsychiatric disorders. The exclusion criteria were as follows: 1) participants with missing identifiable demographic information, such as age and sex; 2) poor imaging quality that was not suitable for analysis. Specifically, for the retinal fundus imaging, images with significant artifacts and blurry structures were excluded; 3) incomplete brain MRI data or retinal imaging data; 4) participants with a diagnosis of cancer history. Finally, a total of 755 adult participants were eligible for analysis.

Brain MRI data were acquired using a GE Healthcare 3.0 T MRI system (General Electric 750W, Milwaukee, Wisconsin, USA) equipped with an 8-channel phased array head coil at Kailuan General Hospital. Total brain volume was calculated based on high-resolution T1-weighted imaging (T1WI) sequences obtained through three-dimensional BRAin VOlume imaging (3D-BRAVO). The acquisition parameters for 3D-BRAVO-T1WI have been previously described in detail [15]. After standardized preprocessing steps, including reorientation, segmentation, and spatial normalization, automated analysis was performed using the CAT 12 toolbox in SPM 12 (http://www.neuro.uni-jena.de) to calculate gray matter and white matter volumes for all subjects. Total brain volume was computed as the sum of the gray and white matter volumes.

**Fig. 1:**
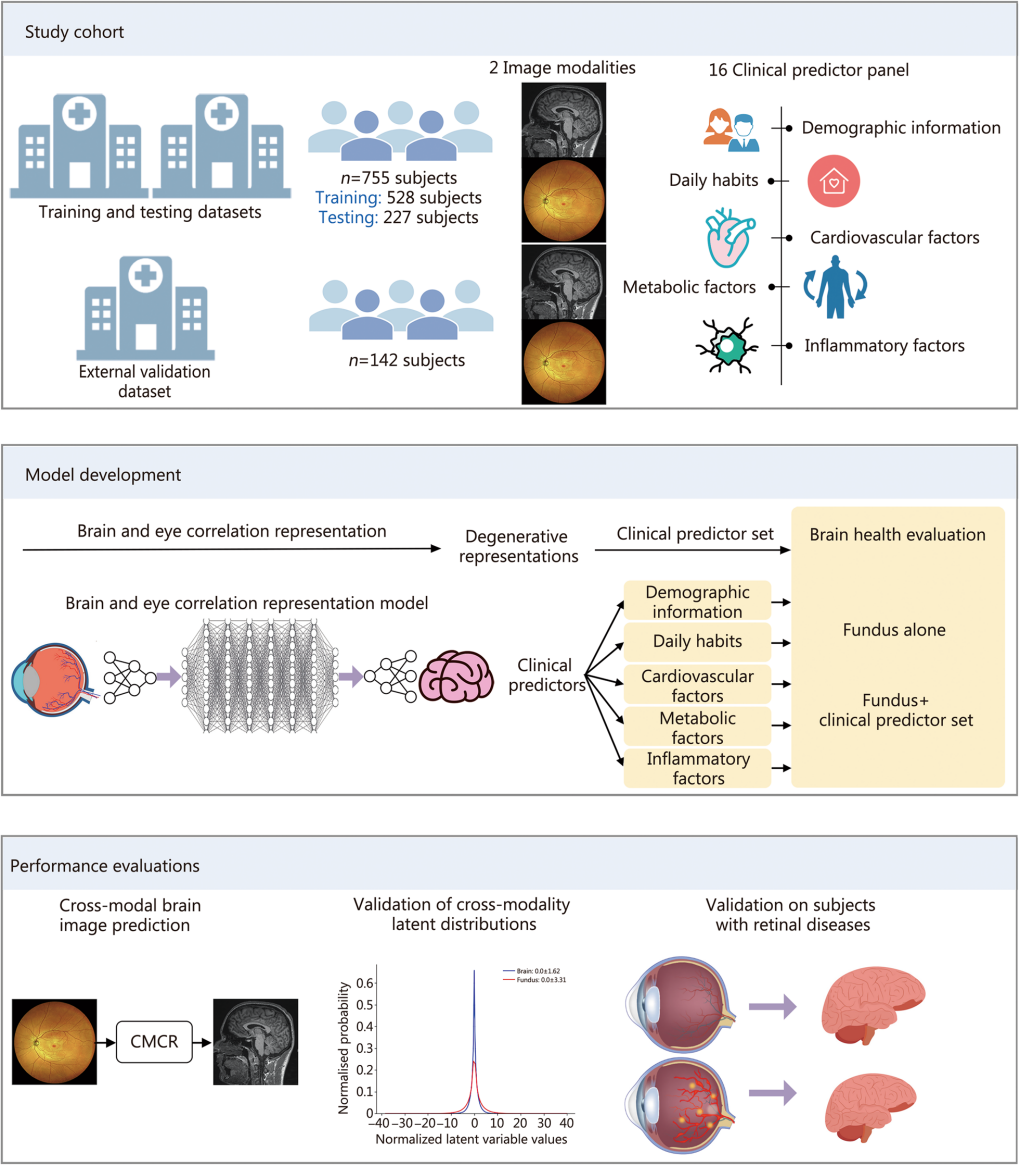
Study overview. CMCR cross-modal correlation representation

**Fig. 2:**
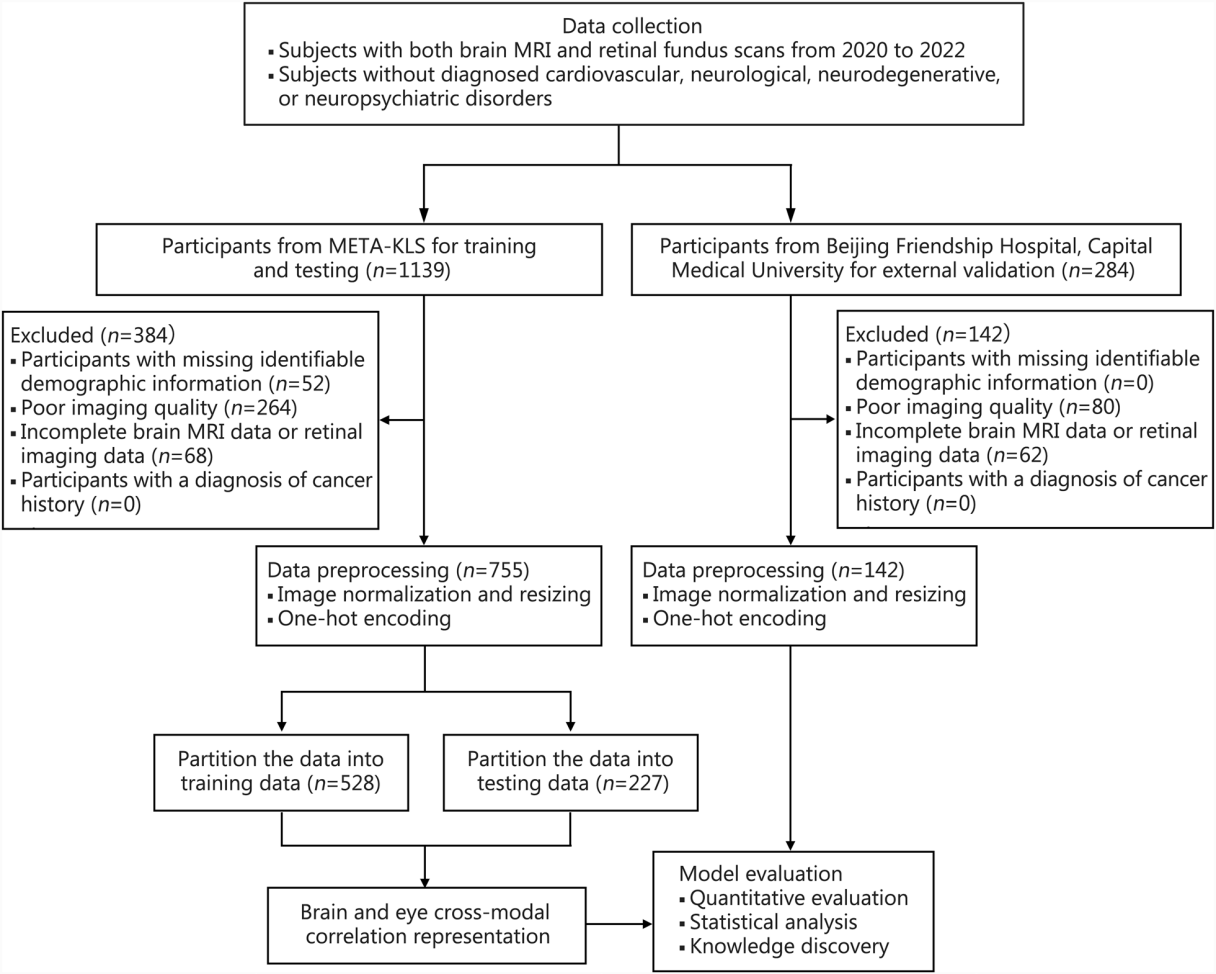
Data processing steps. MRI magnetic resonance imaging

Additionally, to validate the generalizability and clinical applicability of the proposed method, an external validation dataset was obtained from Beijing Friendship Hospital, Capital Medical University. The inclusion and exclusion criteria were consistent with those specified for the META-KLS subjects. There were 142 subjects with both retinal fundus and 3D T1-weighted images, with the same parameters as META-KLS. The age and sex information were also collected. An overview of the study design was illustrated in Fig. 1, and the data processing steps were presented in Fig. 2.

This study has been approved by the Medical Ethics Committee of Kailuan General Hospital (2021002) and Beijing Friendship Hospital, Capital Medical University (2020-P2-155-01). All participants provided written informed consent, and the imaging data utilized in this research were anonymized.

### Details of characteristics

To investigate the impact of individual clinical information on brain volume, the key factors that affect brain health were categorized into 5 types, including demographic information, daily habits, cardiovascular factors, metabolic factors, and inflammatory factors (Additional file 1: Table S1). The multi-type clinical metadata of subjects, collected retrospectively between 2006 and 2018, were utilized in this study for training (70%) and testing (30%) (Additional file 1: Table S2).

### Functional overview of the brain volume quantification system

**Fig. 3:**
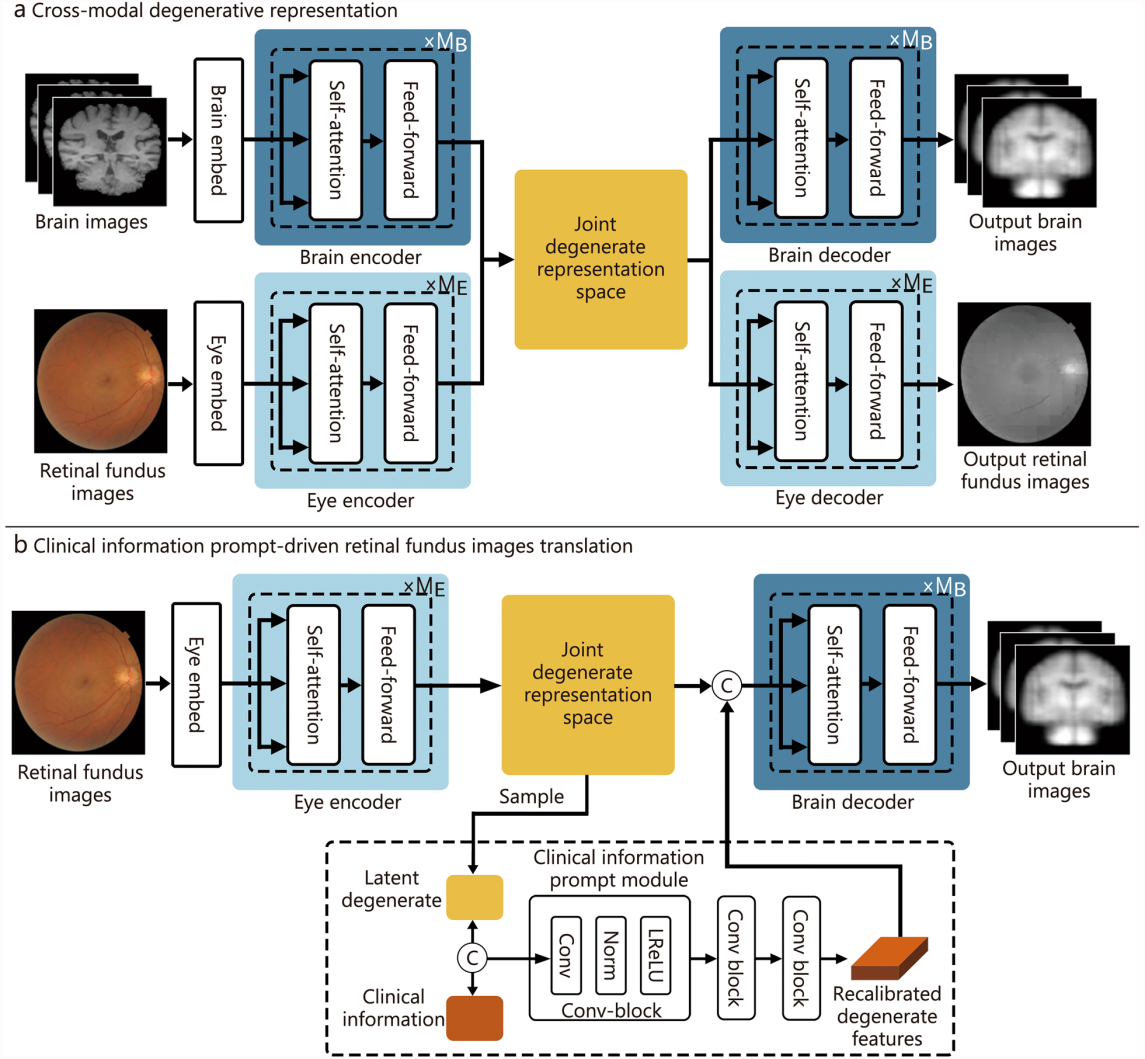
The overall architecture of the proposed brain and eye correlation representation framework. The framework consisted of two main components: the cross-modal degenerative representation network and the clinical information prompt-driven retinal fundus images translation network. The output images represented the synthetic brain images generated from the retinal fundus images. These synthetic brain images were used to estimate brain tissue volume, providing a non-invasive and cost-effective approach to assess brain health. xM_B_ and xM_E_ represent M_B_ and M_E_ identical modules, respectively. Conv convolutional layer, Norm normalization layer, LReLU leaky rectified linear unit

The proposed CMCR network is designed to model the intricate co-degenerative relationships between retinal fundus images and brain MRI data. The architecture consists of two main components: the cross-modal degenerative representation network and the clinical information prompt-driven retinal fundus images translation network, as shown in Fig. 3. The cross-modal degenerative representation network is the core component of the CMCR framework. It is responsible for capturing the degenerative features from both retinal fundus images and brain MRI data and establishing a shared degenerative representation space. The network comprises 3 key modules: the brain encoder, the eye encoder, and the cross-modal degenerative fusion module. The prompt-driven brain degeneration inference model leverages the pre-trained cross-modal degenerative representation network to predict brain images from retinal fundus images. By incorporating clinical information as prompts, this model recalibrates the degenerative features derived from retinal images, thereby enhancing the accuracy of brain volume estimation.

### Comparison methods and performance evaluation metrics

To evaluate the performance of the proposed brain and eye degeneration correlation representation network, several image synthesis methods are utilized for comparison, including multi-channel Variational Autoencoder (mcVAE) [17], Pixel-to-Pixel (Pixel2pixel) [18], transformer-based U-Net (TransUNet) [19], multi-scale transformer network (MT-Net) [20], and residual vision transformer (ResViT) [21]. The implementation details and hyperparameter tuning ranges for each comparative method are summarized in Additional file 1: Table S3.

Three quantitative evaluation metrics are utilized to evaluate the synthesis performance of different models, including: 1) root mean square error (RMSE); 2) peak signal-to-noise ratio (PSNR); and 3) structural similarity index measure (SSIM). The mathematical formulations for RMSE and PSNR are defined as follows:

1 $${\text{RMSE = }}\sqrt {{\frac{1}{{{\text{MN}}}}} \sum\nolimits_{i = 1}^{{\text{M}}} {\sum\nolimits_{j = 1}^{{\text{N}}} {\left( {{\text{X}}_{ij} - {\hat{\text{X}}}_{ij} } \right)} }^{2}}$$

2 $${\text{PSNR = 20log}}_{{{10}}} \frac{{\text{Max(x)}}}{{{\text{RMSE}}}}$$

where $M$ and $N$ represent the size of the images, *i* and $j$ symbolize the coordinate position, $x$ and $\widehat{x}$ represent real voxel and predicted voxel. The PSNR indicates the ratio of the peak signal energy to the average noise energy, and it is inversely proportional to the RMSE. The formulations for SSIM:

3 $${\text{SSIM}} = 1 - \frac{{\left( {2\mu_{x} \mu_{y} + c_{1} } \right)\left( {\sigma_{xy} + c_{2} } \right)}}{{\left( {\mu_{x}^{2} + \mu_{y}^{2} + c_{1} } \right)\left( {\sigma_{x}^{2} + \sigma_{y}^{2} + c_{2} } \right)}}$$

where ${\mu }_{x}$ and ${\mu }_{y}$ represent the mean value in the real and predicted images, ${\sigma }_{x}$ and ${\sigma }_{y}$ represent the standard deviation in the real and predicted images, and ${\sigma }_{xy}$ represents the covariance. ${c}_{1}$ and ${c}_{2}$ represent constants to avoid system errors caused by a denominator of 0. SSIM is a perception-based computational model that focuses on the fuzzy changes of the structural information in human perception.

### Statistical analysis

All statistical analyses were performed using Python (v 3.8.3) with the SciPy library (v 1.10.1) for statistical tests and the NumPy library (v 1.22.4) for numerical computations. Continuous variables were presented as mean ± standard deviation (SD) for normally distributed data or median (interquartile range, IQR) for non-normal distributions, with normality assessed using Shapiro-Wilk tests. Categorical variables were reported as *n* (%).

To comprehensively evaluate the performance of the proposed CMCR framework and assess the statistical significance of improvements over comparative methods, multiple statistical analyses were conducted. Quantitative metrics, including RMSE, PSNR, and SSIM, were computed to systematically assess the accuracy and quality of synthetic brain images. For brain volume estimation, both absolute differences (in cm^3^) and relative errors (percentage difference from ground truth) were reported to provide a detailed evaluation. Paired Student’s *t*-test (or Wilcoxon signed-rank tests for non-normal data) were applied to compare the CMCR framework against each baseline method under identical experimental conditions, including scenarios with and without integrated clinical information. Before conducting paired Student’s *t*-test, we confirmed that 1) observations were matched (same subjects under CMCR or other comparing methods and baseline conditions) and 2) paired differences were normally distributed (Shapiro-Wilk test, *P* > 0.05).

Pearson correlation coefficients (*r*) were calculated to quantitatively assess the linear relationship between the predicted and actual brain volumes in the external validation dataset. Bland-Altman plots were used to visually evaluate the agreement between predicted and actual brain volumes, as well as to identify any potential systematic bias or limits of agreement. *P* < 0.05 (two-tailed) was considered statistically significant.

## Results

### Cross-modal brain image synthesis

The quantitative metrics for cross-modal brain image synthesis from retinal fundus images using this method and other comparative methods are shown in Table 1. Specifically, the proposed CMCR framework achieves an RMSE of 98.23, a PSNR of 35.78 dB, and a SSIM of 0.64. CMCR achieves statistically significant improvements across all metrics, demonstrating superior performance in cross-modal brain image generation from retinal fundus images. It consistently outperforms existing methods in accuracy, signal-to-noise ratio, and structural similarity.

**Table 1:** Comparative performance analysis of cross-modal brain image synthesis (mean ± SD, *n* = 227)

Methods	RMSE	PSNR (dB)	SSIM
mcVAE	232.67 ± 43.67**	28.42 ± 2.38**	0.42 ± 0.08**
Pixel2pixel	210.23 ± 39.48**	29.35 ± 2.06**	0.50 ± 0.06**
TransUNet	198.55 ± 38.61**	30.12 ± 1.92**	0.53 ± 0.05**
MT-Net	172.63 ± 30.28**	32.54 ± 1.27**	0.54 ± 0.05**
ResViT	165.58 ± 27.87**	33.56 ± 1.10*	0.58 ± 0.04**
CMCR	98.23 ± 18.92	35.78 ± 1.01	0.64 ± 0.04

*P < 0.05, **P < 0.01 vs. CMCR. *RMSE* root mean square error, *PSNR* peak signal-to-noise ratio, *SSIM* structural similarity index measure, *mcVAE* multi-channel Variational Autoencoder, *Pixel2pixel* Pixel-to-Pixel, *TransUNet* transformer-based U-Net, *MT-Net* multi-scale transformer network, *ResViT* residual vision transformer, *CMCR* cross-modal correlation representation

**Fig. 4:**
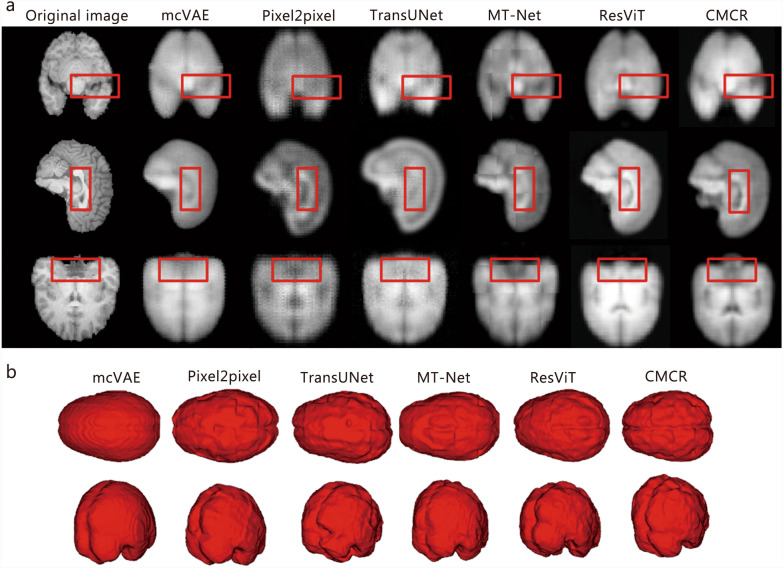
Synthetic brain images in 2D **a** and 3D **b** generated from retinal fundus images using the CMCR method and other comparative methods. The red boxes highlight the regions exhibiting significant improvements achieved by the CMCR framework. 2D two-dimensional, 3D three-dimensional, mcVAE multi-channel Variational Autoencoder, Pixel2pixel Pixel-to-Pixel, TransUNet transformer-based U-Net, MT-Net multi-scale transformer network, ResViT residual vision transformer, CMCR cross-modal correlation representation

The 2D and 3D comparisons of the predicted brain images generated by various methods are presented in Fig. 4. All 6 methods can roughly predict the brain outline. However, more precise contours and local details are significantly compromised by the other 5 comparative methods. The proposed CMCR network not only accurately delineates the cerebral sulcus but also generates rich texture details, which are essential for brain volume calculation. Thus, these quantitative results, in conjunction with superior 2D and 3D visualizations, provide strong evidence that the CMCR framework more accurately captures the latent structural correlations between the fundus and the brain.

### Validation of cross-modality latent distributions

In addition to quantitatively evaluating the quality of the generated images, the discrepancies in the latent degenerate features of the brain and eye in the joint degenerate space are also evaluated. To validate that the latent distributions of brain and retinal fundus images are effectively encoded by the proposed network, the distributions are illustrated in Additional file 1: Fig. S1. The results suggest that brain and retinal fundus images show consistent distributions in latent degenerate space, thereby validating the feasibility of quantifying brain volumes using retinal fundus images.

The peak differences in the distribution of brain and retinal fundus images in Additional file 1: Fig. S1 arise from the inherent differences in the modality-specific characteristics of the two imaging types. While both brain MRI and retinal fundus images are utilized to assess degenerative changes, they capture distinct aspects of tissue structure and pathology. The brain MRI provides high-resolution 3D structural information, capturing detailed volumetric and morphological alterations in brain tissues. The degenerative features extracted from brain MRI are typically more globally distributed across the brain, leading to a broader and more uniform distribution in the latent space. In contrast, the 2D retinal fundus images primarily capture microvascular and structural changes in the retina. The degenerative features derived from retinal fundus images are often more localized, focusing on specific regions such as the optic disc or retinal vasculature. This localized nature can result in a more concentrated distribution in the latent space. These differences in the spatial and structural characteristics of the two modalities contribute to the observed peak differences in their latent space distributions. However, despite these differences, the shared degenerative representation space established by the CMCR framework effectively captures the underlying degenerative relationships between the fundus and brain, as evidenced by the consistent alignment of their distributions.

### Cross-modal brain volume quantification

The brain volumes derived from the predicted brain images are calculated and utilized as an important metric to characterize brain health status. In the experiments, the differences between the estimated brain volumes and the ground truths are shown in Table 2. The average difference between estimated brain volumes derived from generated brain images and the actual brain volumes is 61.36 cm^3^, with a relative error of 4.54%. Compared with other methods, our proposed method exhibits the smallest error in predicting brain volumes. The quantitative evaluation results are consistent with the visualization results in Fig. 4.

**Table 2:** Differences between the estimated brain volumes and the ground truths (mean ± SD, *n* = 227)

Clinical information	Absolute differences (cm^3^)						Relative errors (%)					
	mcVAE	Pixel2pixel	TransUNet	MT-Net	ResViT	CMCR	mcVAE	Pixel2pixel	TransUNet	MT-Net	ResViT	CMCR
N/A	144.26 ± 26.32**	132.57 ± 26.47**	119.25 ± 21.47**	88.26 ± 13.57**	72.34 ± 11.49**	61.36 ± 9.87	10.67 ± 1.95**	9.80 ± 1.96**	8.82 ± 1.59**	6.53 ± 1.00**	5.35 ± 0.85**	4.54 ± 0.73
With demographic information	137.58 ± 24.37**	123.54 ± 22.54**	116.51 ± 17.54**	85.34 ± 12.34**	70.21 ± 10.92**	59.57 ± 9.24	10.17 ± 1.80**	9.13 ± 1.67**	8.61 ± 1.30**	6.31 ± 0.91**	5.19 ± 0.81**	4.40 ± 0.68
With daily habits	129.76 ± 20.14**	115.49 ± 19.84**	110.24 ± 14.23**	78.21 ± 9.34**	67.26 ± 9.84*	57.93 ± 7.62	9.59 ± 1.49**	8.54 ± 1.47**	8.15 ± 1.05**	5.78 ± 0.69**	4.97 ± 0.73*	4.28 ± 0.56
With cardiovascular factors	127.54 ± 18.67**	113.69 ± 15.56**	108.34 ± 13.69**	77.22 ± 9.89**	65.64 ± 8.26**	56.98 ± 7.53	9.43 ± 1.38**	8.41 ± 1.15**	8.01 ± 1.01**	5.71 ± 0.73**	4.85 ± 0.61**	4.21 ± 0.56
With metabolic factors	124.78 ± 18.02**	106.28 ± 13.51**	103.25 ± 12.37**	74.32 ± 8.74**	62.63 ± 7.54**	55.75 ± 6.97	9.23 ± 1.33**	7.86 ± 1.00**	7.63 ± 0.91**	5.49 ± 0.65**	4.63 ± 0.56**	4.12 ± 0.52
With inflammatory factors	120.58 ± 13.34**	104.41 ± 12.43**	92.58 ± 10.67**	72.21 ± 8.42**	60.29 ± 8.69*	53.89 ± 6.51	8.92 ± 0.99**	7.72 ± 0.92**	6.84 ± 0.79**	5.34 ± 0.62**	4.46 ± 0.64*	3.98 ± 0.48

The CMCR method has been compared in performance with other methods at every step using paired Student’s *t*-test. ^*^P < 0.05, ^**^P < 0.01 vs. CMCR. The results are reported in absolute differences (cm^3^) and relative errors (%). *N/A* not including any clinical information, *mcVAE* multi-channel Variational Autoencoder, *Pixel2pixel* Pixel-to-Pixel, *TransUNet* transformer-based U-Net, *MT-Net* multi-scale transformer network, *ResViT* residual vision transformer, *CMCR* cross-modal correlation representation

To further investigate the contributions of clinical information to the proposed model, 5 categories of clinical information are incrementally encoded and integrated with the degenerate latent code. It can be observed that as more categories of clinical information are integrated, the estimated brain volumes are closer to the ground truths. With the incremental incorporation of age and sex, daily habits, cardiovascular factors, metabolic factors, and inflammatory factors, the differences in estimated brain volume were reduced by 1.79, 3.43, 4.38, 5.61, and 7.47 cm^3^, respectively. When all of the clinical information was encoded, the difference was decreased to 53.89 cm^3^, with a relative error of 3.98%. Additionally, our method exhibits significantly superior performance over other methods.

**Fig. 5:**
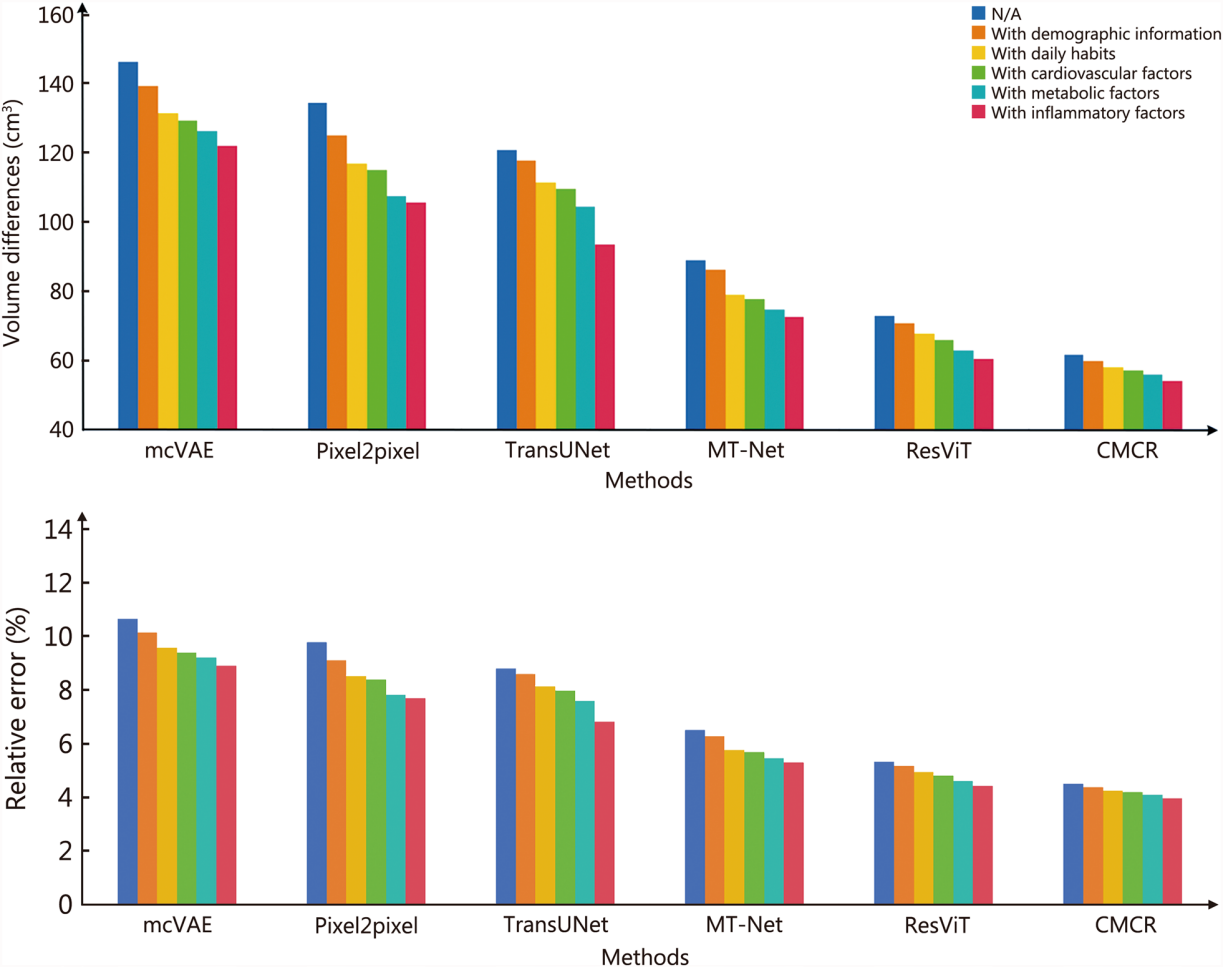
Bar charts illustrating the contributions of various types of clinical information in predicting brain volumes using retinal fundus images. N/A not including any clinical information, mcVAE multi-channel Variational Autoencoder, Pixel2pixel Pixel-to-Pixel, TransUNet transformer-based U-Net, MT-Net multi-scale transformer network, ResViT residual vision transformer, CMCR cross-modal correlation representation

The bar charts in Fig. 5 illustrate the contributions of various types of clinical information in predicting brain volumes using retinal fundus images. It is evident that age and sex, and daily habits exert a more significant influence compared to other clinical factors. More importantly, the predictive results have achieved a considerably high accuracy without the inclusion of invasive examination data or less commonly used parameters. The Bland-Altman plots presented in Additional file 1: Fig. S2 illustrate the differences between estimated brain volumes and ground truths, with and without the incorporation of clinical information.

### Validation on subjects with retinal diseases

To evaluate the generalizability of the proposed CMCR across diverse clinical conditions, brain volume estimation results for subjects diagnosed with common retinal diseases were presented in Table 3 and Additional file 1: Fig. S3. Specifically, the retinal diseases diagnosed by ophthalmologists included age-related macular degeneration, fundus changes in high myopia, diabetic retinopathy, vitreoretinal interface disorders, and retinal microvascular abnormalities. For subjects with normal fundus images, the CMCR method achieves the lowest brain volume estimation error (48.96 cm^3^), outperforming all comparative methods. For subjects with abnormal fundus images, our method also achieves the lowest error (60.21 cm^3^). Subjects with retinal diseases exhibit slightly larger brain volume differences compared to those without retinal diseases. Despite the increased differences, the CMCR framework still demonstrates superior performance, highlighting its robustness in handling image variability induced by retinal pathology. The findings provide cutting-edge evidence demonstrating the model’s robust generalizability across a wide spectrum of clinical conditions.

**Table 3:** Quantitative evaluation results obtained from subjects with and without retinal diseases (cm^3^, mean ± SD, *n* = 227)

Subjects condition	mcVAE	Pixel2pixel	TransUNet	MT-Net	ResViT	CMCR
Normal fundus	100.62 ± 12.67**	93.45 ± 14.36**	88.06 ± 9.67**	69.29 ± 7.98**	58.49 ± 6.24*	48.96 ± 5.69
Abnormal fundus	140.38 ± 14.34**	114.56 ± 15.64**	100.68 ± 11.21**	82.20 ± 10.26**	69.92 ± 6.69*	60.21 ± 7.36

The CMCR method has been compared in performance with other methods using paired Student’s *t*-test. ^*^P < 0.05, ^**^P < 0.01 vs. CMCR. *mcVAE* multi-channel Variational Autoencoder, *Pixel2pixel* Pixel-to-Pixel, *TransUNet* transformer-based U-Net, *MT-Net* multi-scale transformer network, *ResViT* residual vision transformer, *CMCR* cross-modal correlation representation

### External validation

External validation experiments are conducted using an external dataset collected from Beijing Friendship Hospital, Capital Medical University. The dataset includes 142 subjects with matched retinal fundus and brain T1 images. Only age and sex information are available for analysis.

The quantitative evaluation results on the external dataset are presented in Table 4. Due to variations in the quality of retinal fundus images from different centers, the discrepancy between estimated brain volume and the actual brain volume has increased (Additional file 1: Fig. S4). Nevertheless, the proposed CMCR still exhibits the best performance and generalizability in estimating brain volumes.

**Table 4:** Quantitative evaluation results obtained from the external validation set (*n* = 142)

Methods	Brain volume differences (cm^3^, mean ± SD)	Pearson’s *r*	*P*-value
mcVAE			
N/A	183.28 ± 28.63**	0.6299	0.007
With demographic information	175.24 ± 25.64**	0.6598	0.007
Pixel2pixel			
N/A	176.54 ± 24.36**	0.6785	0.006
With demographic information	162.76 ± 22.94**	0.6951	0.006
TransUNet			
N/A	146.68 ± 19.34**	0.7084	0.006
With demographic information	139.62 ± 17.37**	0.7121	0.008
MT-Net			
N/A	106.74 ± 15.97**	0.7326	0.008
With demographic information	95.47 ± 14.09**	0.7569	0.009
ResViT			
N/A	99.32 ± 12.67**	0.7463	0.008
With demographic information	90.91 ± 11.93**	0.7782	0.008
CMCR			
N/A	77.14 ± 9.82	0.8274	–
With demographic information	71.24 ± 8.20	0.8451	–

The performance of our proposed method is compared against that of other methods using paired Student’s *t*-test, both with and without the inclusion of age and sex. ^*^P < 0.01 vs. CMCR. “-” indicates that statistical comparison is not applicable because the CMCR model serves as the reference method in all paired *t*-tests. *N/A* not including demographic information, *mcVAE* multi-channel Variational Autoencoder, *Pixel2pixel* Pixel-to-Pixel, *TransUNet* transformer-based U-Net, *MT-Net* multi-scale transformer network, *ResViT* residual vision transformer, *CMCR* cross-modal correlation representation

To evaluate the stability of the proposed method, Pearson correlation between the predicted and actual brain volumes, as well as a paired Student’s *t*-test between our method and other methods, is conducted on the external dataset. It is noteworthy that the proposed CMCR exhibits the highest Pearson’s *r* values among all methods, indicating robust correlations between predicted and actual brain volumes. In summary, all analyses suggest that the proposed CMCR holds promise as a reliable and cost-effective clinical tool for assessing brain volumes through retinal fundus images.

### Time complexity analysis

The time complexity is analyzed to evaluate the computational efficiency and scalability of our CMCR framework. The framework consists of several key components, each contributing to the overall time complexity.

#### Brain encoder and eye encoder

Both the brain encoder and eye encoder utilize a Vision Transformer (ViT) architecture. The time complexity is primarily determined by the number of patches and the dimensionality of the embeddings. Each transformer layer involves self-attention and feed-forward operations. The time complexities for self-attention are $O((N^{B})^{2}\cdot D)$ and $O((N^{E})^{2}\cdot D)$, respectively, where $D$ is the dimensionality of the embeddings. For ${M}_{B}$ and ${M}_{E}$ transformer layers in brain and eye encoders, the total time complexities are $O({M}_{B}\cdot ((N^{B})^{2}\cdot D+{N}^{B}\cdot {D}^{2}))$ and $O({M}_{E}\cdot ((N^{E})^{2}\cdot D+{N}^{E}\cdot {D}^{2}))$, respectively.

#### Cross-modal degenerative fusion module

The cross-modal degenerative fusion module integrates the degenerative features from the brain encoder and eye encoder using a cross-modal attention mechanism. The cross-modal attention mechanism involves computing attention scores between the brain and eye features. The time complexity for cross-modal attention is $O({N}^{B}\cdot {N}^{E}\cdot D)$, where ${N}^{B}$ and ${N}^{E}$ are the number of patches for the brain and eye images, respectively.

#### Brain decoder and eye decoder

The brain decoder and eye decoder employ transformer-based modules to map high-level degenerative representations to lower semantic representations and perform low-level reconstruction. Similar to the encoders, the time complexities for the transformer layers in brain and eye decoders are $O({M}_{B}\cdot ((N^{B})^{2}\cdot D+{N}^{B}\cdot {D}^{2}))$ and $O({M}_{E}\cdot ((N^{E})^{2}\cdot D+{N}^{E}\cdot {D}^{2}))$, respectively. The reconstruction process involves upsampling and convolutional operations, with the time complexities of $O({N}^{B}\cdot D)$ and $O({N}^{E}\cdot D)$, respectively. The overall time complexity of our CMCR framework are the sum of the time complexities of its components. The overall time complexity can be approximated as:

$$O( {M_{B} \cdot ( {(N^{B} )^{2} \cdot D + N^{B} \cdot D^{2} } ) + M_{E} \cdot ( {(N^{E} )^{2} \cdot D + N^{E} \cdot D^{2} } ) + N^{B} \cdot N^{E} \cdot D + N^{B} \cdot D + N^{E} \cdot D} )$$

where ${M}_{B}$ and ${M}_{E}$ is the number of transformer layers in the brain and eye encoders/decoders, respectively; ${N}^{B}$ and ${N}^{E}$ is the number of patches for the brain and eye images, respectively, and $D$ is the dimensionality of the embeddings.

In addition to demonstrating robust performance across diverse clinical settings, our time complexity analysis provides important insights into the computational feasibility of the CMCR framework. By analytically decomposing the time complexity of each core component, specifically, the ViT-based brain and eye encoders/decoders, as well as the cross-modal attention fusion module, we have validated that the model achieves a strong balance between representational capacity and computational efficiency.

This significance is particularly pronounced given the intended application of our model in large-scale population health screening and resource-constrained clinical settings. The use of transformer-based modules, while inherently powerful, is often associated with substantial computational costs. Our detailed complexity evaluation confirms that the CMCR framework remains tractable for practical deployment, facilitating its integration into real-world scenarios where Graphics Processing Unit (GPU) resources may be limited or batch processing is required.

Furthermore, the modular analysis of computational burden provides a roadmap for further optimization and scalability. Future research could explore lightweight model variants, such as hybrid CNN-transformer structures or sparse attention mechanisms, to further reduce latency while maintaining performance.

Collectively, these findings highlight the potential of CMCR not only as a clinically accurate and biologically interpretable system but also as a computationally efficient solution for accessible and cost-effective brain health evaluation.

## Discussion

Brain health is an evolving major public health concern that has attracted growing interest from both the scientific community and broader society. Brain volume measurement for assessing the integrity of the brain’s macrostructure is a critical approach in the regular screening of brain health. In this study, we developed a cross-modal deep learning framework integrating retinal images and brain MR images to learn a combined representation of eye-brain structural correlations. This framework demonstrated that brain MR images can be synthesized across modalities from retinal fundus images, and that retinal fundus images, along with individual clinical metadata, possess significant potential for accurately estimating brain tissue volume. The study presents an innovative, accurate, and cost-effective approach for characterizing brain health status through readily accessible retinal fundus images.

The design and hypothesis of our study are determined based on the well-established physiological [22], pathological [23], and genetic [12] connections between the eyes and brain. Therefore, we proposed a cross-modal eye-brain degenerative correlation representation model to effectively and quantitatively evaluate the degenerative status of both the eyes and brain. This strategy fully capitalizes on the pathophysiological associations between the eyes and brain while enhancing the interpretability of the results. Specifically, the volume of brain tissue can be accurately estimated based on synthesized brain MR images generated from retinal imaging data. In terms of algorithms, our framework addresses the limitations of previous work by fully leveraging the high-dimensional information embedded within retinal and brain imaging data, rather than focusing exclusively on quantitative features or genetic phenotypes. Specifically, the CMCR framework introduces several innovative aspects compared to existing methods that are widely used for multi-type data integration.

1. Unlike existing methods that handle separate specific imaging data as independent inputs, our framework explicitly models the degenerative interrelationship between retinal and brain imaging modalities. This is accomplished through a Variational Spatial-Transformer Auto-Encoder (VTAE), which captures high-dimensional degenerative features from both retinal fundus and brain images, enabling a more comprehensive and interpretable representation of brain health.
2. We incorporate clinical information as prompts to recalibrate the degenerative features extracted from retinal images. This approach dynamically adjusts the latent representations based on diverse categories of personal information, significantly enhancing the accuracy of brain volume estimation and providing a more individualized assessment of brain health.
3. Multi-modal masked modeling is employed in the training of the proposed cross-modal degenerative representation network, where patches from both retinal and brain images are randomly masked. This technique drives the model to learn robust cross-modal representations by reconstructing the masked regions, enhancing its capability to capture latent degenerative relationships even with incomplete or noisy data.
4. Our framework exhibits robust generalization performance across diverse datasets, as demonstrated by the outcomes of rigorous internal and external validation.

Our systematic work presents numerous advantages over existing research on eye-brain joint analysis, providing a significant complement to the exploration of eye-brain correlation. Firstly, we innovatively utilized original retinal fundus images for model training and fully exploited the high-dimensional information inherent in retinal imaging and the interrelationship between retinal and brain structures. This tailored model extends beyond the conventional focus on quantitative features or genetic phenotypes, representing a significant advancement in current eye-brain joint analysis. Secondly, the research represents a substantial advancement in the evaluation of brain health across a broader demographic. Although brain tissue atrophy is widely recognized as an early indicator of neurological disorders and neurodegenerative diseases [24–26], MRI-based measurements exhibit limited generalizability in resource-limited settings, such as rural areas and aerospace exploration, where access to advanced neuroimaging examinations, real-time radiological interpretation, and quantitative analysis remains restricted. Therefore, the study introduces an innovative approach to assess the status of brain health without the necessity of neuroimaging data acquisition, thus facilitating the regular screening of brain health in the general population while minimizing medical financial burdens. The estimated smaller brain volume may serve as an early alert for further medical consultation, facilitating timely referrals for additional examinations and promoting the early diagnosis and treatment of neurological diseases. Thirdly, we also conducted a comprehensive analysis of the model’s performance in subjects with retinal diseases. The robustness of prediction results demonstrated the reliable generalizability of the proposed model across diverse clinical conditions, thereby enhancing its clinical applicability and proficiency in addressing various common challenges encountered in clinical settings. Lastly, the participants of the KLS were recruited across 11 hospitals in Tangshan city, located at the central area of the Bohai Sea Gulf region [15]. The findings can be effectively generalized to diverse populations within the Western Pacific regions.

There were two primary considerations for incorporating multi-category clinical information to refine the prediction model. Firstly, a substantial body of prior research has demonstrated that personal metadata, including physical examination results [27–29], lifestyle factors [30–32], cardiovascular factors [33–35], metabolic indicators [36–38], and inflammatory markers [39], are significantly correlated with brain health. Integrating this medical knowledge into the model is not only a crucial enhancement for interdisciplinary research in medicine and engineering but also an essential prerequisite for the application of the model in clinical settings. Secondly, diverse categories of clinical data were incrementally encoded into the proposed model, progressing from the most readily available to less commonly used factors. Specifically, basic demographic information such as chronological age and sex was prioritized for inclusion. Subsequently, lifestyle factors such as smoking and alcohol consumption can be easily obtained through questionnaire interviews. Cardiovascular factors, such as blood pressure, lipid profiles, and fasting blood glucose levels, are assessed through invasive or non-invasive methods during physical examinations by well-trained clinicians. Finally, metabolic and inflammatory markers, including creatinine, uric acid, and neutrophils, are less frequently acquired in physical examinations or in population-based cohorts and were therefore encoded in the final step. The experimental results demonstrated that the sequential integration of subject metadata progressively improved prediction accuracy and achieved more accurate estimates of brain volume. The outcome validated the significantly positive contribution of multi-category clinical information to our model and validated the research hypothesis that the volumes of brain tissues can be accurately estimated using retinal fundus images together with clinical information. Therefore, the integration of diverse categories of clinical information demonstrates substantial value in enabling more precise assessment of individual brain health.

Specifically, the availability and accessibility of clinical information need to be carefully considered for model interpretation in clinical settings. As illustrated in Table 2, brain tissue volume could be estimated with considerable accuracy using age, sex, and lifestyle information, in the absence of data from invasive assessments or less commonly examined parameters. This finding significantly enhances the practicality of implementing our proposed system into clinical settings for routine brain health assessments.

For the validation set, only age and sex are available, while the other clinical features used in training are missing. Theoretically, incorporating the full set of clinical data could achieve better performance. However, considering the practicality of future clinical applications, the clinical features that can be included in practical applications may be limited. Moreover, it was significantly difficult to obtain an optimal validation dataset with the full set of clinical data. Since age and sex informations were readily available, we strategically tried to incorporate only the information in the validation analysis. The proposed model demonstrated reliable high accuracy within the validation dataset, thereby laying the groundwork for future practical implementation.

There are several limitations in our study that we aim to address in future research. First, given the cross-sectional design of this study, our research establishes a shared degenerative representation between retinal fundus images and brain images at the baseline level. Future research should further investigate the predictive capacity of retinal images for the progression of brain atrophy using longitudinal eye-brain imaging data. Such longitudinal validation could provide critical insights into the temporal relationships between retinal and brain degeneration, potentially facilitating the identification of early markers of neurodegeneration and enabling personalized diagnosis and intervention. Second, while brain macrostructural volume is a crucial neurobiomarker of brain health status [16, 29, 40], it cannot definitively indicate the incidence of any specific neurological diseases. The estimated brain volume results may serve as an alert signal rather than a definitive diagnostic criterion. Moreover, future research is warranted to extend the investigations into distinct neuroanatomical regions, particularly those with significant pathological implications, such as the hippocampus associated with AD. Third, although the training and testing datasets utilized in this study exhibit differences in demographic information such as age and sex, these clinical variables have been adjusted during the model development. External validation was also conducted to examine the impact of dataset discrepancies on model performance. Lastly, it may also be necessary to develop improved algorithms for predicting other subtle neuroimaging markers in asymptomatic individuals, such as the detection of cerebral microbleeds or the quantification of white matter hyperintensity that is closely associated with cerebral small vessel disease [41, 42].

## Conclusions

In this study, we proposed a novel tailored cross-modal eye and brain degenerative correlation representation framework to capture the latent degenerative relationships between the eyes and brain, and estimate the brain tissue volume from retinal fundus images and clinical information. The study provides an innovative, reliable, and cost-effective approach to accurately characterize brain health status through readily accessible retinal fundus images.

## Supplementary Information

**Additional file** **1.** **Table S1** The categories of clinical information. **Table S2** Comparison of clinical characteristics between training and testing datasets (*n* = 755). **Table S3** The implementation details of the comparison methods. **Fig. S1** Comparison of the latent space variance: distribution of the latent variables obtained using the proposed model on retinal fundus images and brain images. **Fig. S2** Bland-Altman plots for estimated brain volumes and ground truths, without **a** and with **b** the incorporation of clinical information. **Fig. S3** Scatter plots of the estimated brain volume and the actual brain volume for subjects with normal and abnormal fundus conditions. **Fig. S4** Bland-Altman plots for estimated brain volumes and ground truths, without **a** and with **b** the incorporation of clinical information on the external validation set.

## Data Availability

Clinical data will be available for other research groups whose proposed use of the data has been approved by an independent review committee identified for this purpose. Requests for data should be directed to the principal investigator, Dr. Zhen-Chang Wang (cjr.wzhch@vip.163.com).

## Abbreviations

2D: Two-dimensional
3D: Three-dimensional
3D-BRAVO: Three-dimensional BRAin VOlume imaging
AD: Alzheimer’s disease
CMCR: Cross-modal correlation representation
GT: Ground truth
KLS: KaiLuan Study
MCI: Mild cognitive impairment
mcVAE: Multi-channel variational autoencoder
META-KLS: Multi-modality MEdical imaging sTudy bAsed on the KaiLuan Study
MRI: Magnetic resonance imaging
MT-Net: Multi-scale transformer network
Pixel2pixel: Pixel-to-pixel
PSNR: Peak signal-to-noise ratio
ResViT: Residual vision transformer
RMSE: Root mean square error
SD: Standard deviation
SSIM: Structural similarity index measure
T1WI: T1-weighted imaging
TransUNet: Transformer-based U-Net
VTAE: Variational Spatial-Transformer Auto-Encoder
