# Supplementary material for: Clinical information prompt-driven retinal fundus image for brain health evaluation
Source: Mil Med Res. 2025 Aug 6;12:47. doi: 10.1186/s40779-025-00630-2 (PMC12329875; doi:10.1186/s40779-025-00630-2)
Supplement: Supplementary file 1 — Additional file 1. Table S1 The categories of clinical information. Table S2 Comparison of clinical characteristics between training and testing datasets (n = 755). Table S3 The implementation details of the comparison methods. Fig. S1 Comparison of the latent space variance: distribution of the latent variables obtained using the proposed model on retinal fundus images and brain images. Fig. S2 Bland-Altman plots for estimated brain volumes and ground truths, without a and with b the incorporation of clinical information. Fig. S3 Scatter plots of the estimated brain volume and the actual brain volume for subjects with normal and abnormal fundus conditions. Fig. S4 Bland-Altman plots for estimated brain volumes and ground truths, without a and with b the incorporation of clinical information on the external validation set. [file 40779_2025_630_MOESM1_ESM.pdf]

Model architecture

Brain and eye correlation representation model

Firstly, the framework efficiently integrates degenerative properties derived from retinal fundus scans and brain images, offering a comprehensive and personalized perspective that synthesizes features from multi-modal data (cross-modal degenerative representation in **Fig. 3**).

Multi-modal brain and eye Variational Spatial-Transformer Auto-Encoder (VTAE) capture degenerative features from both retinal fundus scans and brain images, while the inference model utilizes the latent degenerate representation to predict brain conditions. The continuous latent space of VTAE further provides an interpretable representation of degenerative characteristics, facilitating the inference of the brain. The latent relationships between the eyes and brain are learned by pre-training the degenerative correlation representation model on paired brain and eye images. Formally, given a pair of brain magnetic resonance imaging (MRI) data, I^B and the corresponding eye images I^E from the same subject, the details of the network pre-training and cross-modal degeneration quantification will be introduced in the following sections.

Cross-modal variational spatial-transformer auto-encoder

The backbone, brain, and eye degeneration correlation representation model $\mathcal{M}_\theta$ comprises 3 components: the brain and eye encoders, which encode the input brain and eye images, respectively, and a cross-modal fusion module that interacts with the extracted degenerative features to construct the latent degenerative representation space.

Brain encoder In this study, the Vision Transformer (ViT) [1] architecture is employed for encoding the brain degenerative features. Specifically, in ViT, a three-dimensional (3D) brain image $I^B \in \mathbb{R}^{L^B \times W^B \times H^B \times C^B}$ is first segmented into patches $p_1^B, p_2^B, \ldots, p_{N^B}^B$, where $L^B \times W^B \times H^B$ is the resolution of the input brain image, C^B is the number of channels, $p_{N^B}^B \in \mathbb{R}^{(P^B)^3 \times C^B}$ and $(P^B)^3$ is the patch resolution. Then, the patches are flattened and linearly projected into patch embeddings via a linear transformation $\mathbb{E}^B \in \mathbb{R}^{(P^B)^3 C^B \times D^B}$, with an additional learnable token embedding $p_{I^B}^B \in \mathbb{R}^{D^B}$ introduced for brain feature aggregation. Then, the brain representations are obtained by summing up the patch embeddings and a learnable one-dimensional (1D) position embedding $\mathbb{E}_{pos}^B \in \mathbb{R}^{(N^B+1) \times D^B}$:

$$X^B = [p_{I^B}^B; p_1^B \mathbb{E}^B; p_2^B \mathbb{E}^B; \ldots; p_{N^B}^B \mathbb{E}^B] + \mathbb{E}_{pos}^B \quad (1)$$

The brain degenerative representation X^B is then input to a transformer model, which consists of M_B transformer layers, to obtain the contextualized brain degenerative representations $H^B = [h_{I^B}^B; h_1^B; h_2^B; \ldots; h_{N^B}^B]$.

Eye encoder Similar architecture is also utilized to obtain the eye degenerative representations. For the input two-dimensional (2D) retinal fundus images $I^E \in \mathbb{R}^{L^E \times W^E \times C^B}$, similar procedures are conducted. Specifically, the image $I^E \in \mathbb{R}^{L^E \times W^E \times C^E}$ is segmented into patches $p_1^E, p_2^E, \ldots, p_{N^E}^E$, where $L^E \times W^E$ is the resolution of the input eye image, C^E is the number of eye channels, $p_{N^E}^E \in \mathbb{R}^{(P^E)^2 \times C^E}$ and $(P^E)^2$ is the patch resolution. Then, the patches are flattened and linearly projected into patch embeddings via a linear transformation $\mathbb{E}^E \in \mathbb{R}^{(P^E)^2 C^E \times D^E}$, with an additional learnable token embedding $p_{I^E}^E \in \mathbb{R}^{D^E}$ introduced for eye feature aggregation. Then, the eye representations are obtained by summing up the patch embeddings and a learnable 1D position embedding $\mathbb{E}_{pos}^E \in \mathbb{R}^{(N^E+1) \times D^E}$:

$$X^E = [p_{I^E}^E; p_1^E \mathbb{E}^E; p_2^E \mathbb{E}^E; \ldots; p_{N^E}^E \mathbb{E}^E] + \mathbb{E}_{pos}^E \quad (2)$$

Similarly, the contextualized eye degenerative representations $H^E = [h_{I^E}^E; h_1^E; h_2^E; \ldots; h_{N^E}^E]$ are acquired by inputting X^E to a transformer model.

Cross-modal degenerate fusion module Utilizing the contextualized degenerative representations derived from brain and eye images, a cross-modal attention fusion module is established to capture the degenerative relationships between the brain and eyes. In detail, the cross-modal attention fusion module consists of two transformer models, each of which is a stack of N_m transformer layers. In each transformer layer, there are 3 sub-layers: a self-attention sub-layer, an across-attention sub-layer, and a feed-forward sub-layer. The attention mechanism is applied in the self-attention and cross-attention sub-layers, which is defined as:

$$ATTN(Q, K, V) = softmax(\frac{QK^T}{\sqrt{d_k}}) \cdot V \quad (3)$$

where d_k is the dimension of K. In the self-attention sub-layer, the modality-specific degenerate representations interact within the modality:

$$H^{BS} = ATTN(H^B, H^B, H^B) \quad (4)$$

$$H^{ES} = ATTN(H^E, H^E, H^E) \quad (5)$$

In the cross-attention sub-layer, the modality-specific degenerative representations interact across modalities to integrate cross-modal degenerative information into their representations:

$$H^{BE} = ATTN(H^{BS}, H^{ES}, H^{ES}) \tag{6}$$

$$H^{EB} = ATTN(H^{ES}, H^{BS}, H^{BS}) \tag{7}$$

Subsequently, H^{BE} and H^{EB} are input to the feed-forward sub-layer to obtain the multi-modal brain degenerative representations $R^B = [r_{I^B}^B; r_1^B; r_2^B; \ldots; r_{N^B}^B]$ and eye degenerative representations $R^E = [r_{I^E}^E; r_1^E; r_2^E; \ldots; r_{N^E}^E]$. To map the degenerative representations from brain and eye into a shared degenerative space, multi-modal masked modeling is employed. Specifically, during pre-training, the patches of the two modalities are masked with a ratio, the remained patches are fed into the modal-specific encoder and cross-modal degeneration fusion module to obtain the multi-modal brain degenerative representations R^B and eye degenerative representations R^E. Then, modality-specific decoders are established to reconstruct the brain and eye images. For both the brain and eye decoders, transformer-based modules are employed to map the high-level degenerative representations to the lower semantic representations and perform low-level reconstruction.

Objective function

Given a pair of brain images I^B and eye image I^E, the modality-specific degenerative representations X^B and X^E are obtained by the brain encoder and eye encoder, respectively. Then, the cross-modal degenerative fusion module is introduced to acquire the cross-modal degeneration correlation representations R^B and R^E. Finally, the modality-specific brain and eye decoders are responsible for reconstructing the brain image $I^{B'}$ and eye image $I^{E'}$, respectively. The reconstruction loss is calculated using the mean squared error (MSE) between the reconstructed and original images in pixel space, which is computed as:

$$L_B = \frac{1}{T}\sum_{i=1}^{T}\left|I^B - I^{B'}\right|^2 \tag{8}$$

$$L_E = \frac{1}{T}\sum_{i=1}^{T}\left|I^E - I^{E'}\right|^2 \tag{9}$$

where T is the number of brain and eye image pairs. Besides the reconstruction loss, Kullback-Leibler (KL) divergence loss is employed to mitigate the distance between the prior and posterior distributions of the latent space. The posterior distribution is presumed to follow a standard normal distribution denoted as $\mathcal{N}(0,1)$. The overall loss function is calculated as:

$$\mathrm{L} = \lambda_{\mathrm{B}} L_B + \lambda_{\mathrm{E}} L_E + \lambda_{\mathrm{KL}} L_{KL} \tag{10}$$

where λ_B, λ_E, and λ_{KL} are balancing parameters.

Specifically, KL divergence loss and MSE-based reconstruction loss are used to enforce the similarities between the reconstructed data and the original data at the pixel level and the encoded

latent variable distribution and the prior distribution.

Clinical information prompt-driven retinal fundus images translation

Subsequently, the downstream task-specific network is developed to achieve cross-modal degeneration prediction, i.e., brain volume estimation.

Based on the pre-trained cross-modal brain and eye correlation representation model, the brain image can be predicted from a given retinal fundus image with the pre-trained eye encoder and a fine-tuned brain decoder on the paired brain and eye dataset. Thus, brain health can be assessed by calculating the brain volume based on the predicted brain images. Furthermore, in addition to the degenerative features obtained from the retinal fundus images, diverse ranges of clinical information are also highly correlated with brain health, including age and sex, daily habits [2-4], cardiovascular factors [5-7], metabolic factors [8-10], and inflammatory factors [11]. Thus, the clinical information is encoded via prompting learning to recalibrate the degenerative features obtained from retinal images.

Specifically, the clinical information is encoded using the proposed clinical information prompt module, which comprises multiple convolutional layers as illustrated in **Fig. 3**. Before encoding, the multiple clinical information is preprocessed to ensure consistency and compatibility with the model, which includes normalizing continuous variables and one-hot encoding categorical variables. The preprocessed clinical information is subsequently passed through a series of convolutional layers to extract relevant features. Each convolutional layer applies a set of learnable filters to the input data, capturing underlying patterns and relationships within the clinical information. The output of the convolutional layers is a set of feature maps that represent the encoded clinical information. To ensure compatibility with the latent degenerate features, the feature maps are then processed through a fully connected layer that reduces their dimensionality, resulting in a compact representation of the clinical information suitable for integration with the latent degenerate features. The encoded clinical information is then concatenated with the latent degenerate features extracted from retinal fundus images along the feature dimension to create a combined feature vector that incorporates both structural information from the retina and contextual information from the clinical data. Subsequently, the combined feature vector is passed through a series of fully connected layers that recalibrate the latent degenerate features based on the encoded clinical information. This recalibration process adjusts the weights of the latent features to reflect the influence of the clinical factors, thereby

enhancing the model's capacity to generate accurate synthetic brain images. Finally, the recalibrated features are fed into the brain decoder to map high-level degenerative representations to lower semantic representations and perform low-level reconstruction of the brain images.

With the pre-trained eye encoder and joint degenerate representation space, latent degenerative features are extracted and integrated with clinical information prompts. The concatenation of the recalibrated degenerative features and the original degenerative features was input to the brain decoder to generate the corresponding brain images. In the clinical information prompt-driven brain image synthesis network, the recalibrated degraded features are concatenated with the original degraded features rather than replacing them. This design is motivated by the necessity to preserve the original information contained in the retinal fundus images while enhancing the feature representation with clinical information adjustments. The concatenation operation allows the model to leverage both the original and recalibrated features, creating a richer and more comprehensive representation that improves the accuracy and robustness of brain image synthesis. Additionally, this approach provides greater flexibility in feature integration and improves the model's ability to adapt to variations in input data quality and completeness.

Based on the generated brain images, brain volume can be estimated using corresponding retinal fundus images as input data. In general, the cross-modal correlation representation (CMCR) network models the correlation between retinal images and brain volume through the following steps: 1) the brain and eye encoders extract degenerative features from brain and retinal fundus images, respectively; 2) the cross-modal degenerative fusion module integrates the degenerative features from both modalities, establishing a shared degenerative representation space that captures the co-degenerative relationships between the brain and retina; 3) the clinical information prompt module recalibrates the degenerative features based on the multiple encoded clinical information; and 4) the brain decoder generates synthetic brain images from the recalibrated degenerative features, enabling the estimation of brain tissue volume.

Implementation details

All experiments were implemented in PyTorch with a single NVIDIA 3090 24 GB GPU. Adam was used as the optimizer, setting the batch size to 16 and the maximum number of epochs to 1000. The initial learning rate was set as 0.0005. For network training and testing, the dataset was randomly split in a ratio of 7:3. During the training process, 10% of the training subjects were randomly

selected as the validation set for hyperparameter tuning. The input size for the retinal fundus image was set to 256 × 256, while the brain T1 image was set to 96 × 256 × 256, respectively. External validation was also performed to validate the robustness of the proposed model.

The training of the brain and eye correlation representation model aims to initialize the network with a robust set of weights by learning the degenerative relationships between retinal fundus images and brain MRI data. The pre-training dataset consists of paired retinal fundus and brain T1 images from the Multi-modality MEdical imaging sTudy bAsed on the KaiLuan Study (META-KLS). The image preprocessing steps include resizing, normalization, and augmentation to increase the diversity of the training data. For network initialization, the brain encoder, eye encoder, and cross-modal degenerative fusion module are initialized with random weights. During pre-training, we employ multi-modal masked modeling to enhance the robustness of the network. Specifically, patches from both retinal and brain images are randomly masked with a predefined ratio of 70%. The network is trained to reconstruct the masked regions using the remaining patches, forcing it to learn robust cross-modal representations. Network optimization is performed using the Adam optimizer with an initial learning rate of 0.0005. The batch size is set to 16, and the maximum number of epochs is 1000.

The training of clinical information prompt-driven retinal fundus images translation aims to adapt the pre-trained network to the specific task of brain volume estimation from retinal fundus images. The fine-tuning dataset consists of retinal fundus images and corresponding brain volumes from the META-KLS. Preprocessing steps are similar to those in the pre-training stage, and clinical information is encoded using the clinical information prompt module. The pre-trained eye encoder and cross-modal degenerative fusion module are retained, while the brain decoder is fine-tuned to generate brain images from the recalibrated degenerative features. The network is fine-tuned using the Adam optimizer with a reduced learning rate of 0.0001. The batch size is set to 16, and the maximum number of epochs is 500. Early stopping is employed with a patience of 30 epochs.

Knowledge discovery in eye-brain correlation

In addition to the quantitative evaluation of brain volume estimation, the proposed CMCR framework facilitates important knowledge discoveries regarding the relationship between retinal imaging and brain health. The experimental validation significantly advances the exploration of the eye-brain correlation by demonstrating the feasibility of assessing macrostructural integrity of the

brain using retinal fundus images. The findings establish a methodological framework that facilitates future investigations into distinct neuroanatomical regions through this non-invasive modality. Furthermore, the present study demonstrates the incremental improvement in brain volume estimation accuracy achieved through the integration of various categories of clinical information into the model. The utilization of clinical metadata not only positively fine-tunes the performance of the model but also provides tailored insights for the precise assessment of individual brain health.

References

1. Dalmaz O, Yurt M, Çukur T. ResViT residual vision transformers for multimodal medical image synthesis. IEEE Trans Med Imaging. 2022,41(10):2598-614.
2. Tan ZS, Spartano NL, Beiser AS, DeCarli C, Auerbach SH, Vasan RS, et al. Physical activity, brain volume, and dementia risk: the Framingham study. J Gerontol A Biol Sci Med Sci. 2017,72(6):789-95.
3. Boller B, Mellah S, Ducharme-Laliberté G, Belleville S. Relationships between years of education, regional grey matter volumes, and working memory-related brain activity in healthy older adults. Brain Imaging Behav. 2017,11(2):304-17.
4. Luhar RB, Sawyer KS, Gravitz Z, Ruiz SM, Oscar-Berman M. Brain volumes and neuropsychological performance are related to current smoking and alcoholism history. Neuropsychiatr Dis Treat. 2013,9:1767-84.
5. Deng YT, Li YZ, Huang SY, Ou YN, Zhang W, Chen SD, et al. Association of life course adiposity with risk of incident dementia: a prospective cohort study of 322,336 participants. Mol Psychiatry. 2022,27(8):3385-95.
6. Chuang SY, Wang PN, Chen LK, Chou KH, Chung CP, Chen CH, et al. Associations of blood pressure and carotid flow velocity with brain volume and cerebral small vessel disease in a community-based population. Transl Stroke Res. 2021,12(2):248-58.
7. Ma Y, Yilmaz P, Bos D, Blacker D, Viswanathan A, Ikram MA, et al. Blood pressure variation and subclinical brain disease. J Am Coll Cardiol. 2020,75(19):2387-99.
8. Hakala JO, Pahkala K, Juonala M, Salo P, Kahonen M, Hutri-Kahonen N, et al. Repeatedly measured serum creatinine and cognitive performance in midlife: the cardiovascular risk in young Finns study. Neurology. 2022,98(22):e2268-e81.
9. Rajagopalan P, Refsum H, Hua X, Toga AW, Jack CR Jr, Weiner MW, et al. Mapping creatinine- and cystatin C-related white matter brain deficits in the elderly. Neurobiol Aging. 2013,34(4):1221-30.
10. Tang X, Song ZH, Cardoso MA, Zhou JB, Simo R. The relationship between uric acid and brain health from observational studies. Metab Brain Dis. 2022,37(6):1989-2003.
11. Jiang L, Cai X, Yao D, Jing J, Mei L, Yang Y, et al. Association of inflammatory markers with cerebral small vessel disease in community-based population. J Neuroinflammation. 2022,19(1):106.

Table S1 The categories of clinical information

**Category**	**Clinical information**
Demographic information	Age and sex
Daily habits	Smoking, alcohol drinking, educational level, and physical exercise
Cardiovascular factors	Blood pressure, fasting blood glucose, blood lipids, body mass index, and waist-to-hip ratio
Metabolic factors	Creatinine and uric acid
Inflammatory factors	Neutrophils

Table S2 Comparison of clinical characteristics between training and testing datasets (*n* = 755)

**Characteristics**	**Training datasets (*n* = 528)**	**Testing datasets (*n* = 227)**	***P*-value**
Age (years, mean ± SD)	54.41 ± 10.26	47.92 ± 9.82	< 0.001
Female [*n* (%)]	231 (43.75)	141 (62.11)	< 0.001
Higher education [*n* (%)]	306 (57.95)	137 (60.35)	0.540
Physical exercise [*n* (%)]	259 (49.05)	127 (55.95)	0.082
Non-smokers [*n* (%)]	258 (48.86)	80 (35.24)	0.001
No alcohol consumption [*n* (%)]	347 (65.72)	132 (58.15)	0.048
Cognitive function within normal ranges [*n* (%)]	254 (48.11)	97 (42.73)	0.175
Body mass index within normal ranges [*n* (%)]	329 (62.31)	137 (60.35)	0.612
Waist circumference within normal ranges [*n* (%)]	376 (71.21)	140 (61.67)	0.010
Waist-to-hip ratio within normal ranges [*n* (%)]	339 (64.20)	135 (59.47)	0.217
Neutrophil within normal ranges [*n* (%)]	500 (94.70)	214 (94.27)	0.814
Creatinine within normal ranges [*n* (%)]	506 (95.83)	215 (94.71)	0.496
Uric acid within normal ranges [*n* (%)]	420 (79.55)	186 (81.94)	0.449
Fasting blood glucose within normal ranges [*n* (%)]	427 (80.87)	194 (85.46)	0.130
Blood pressure within normal ranges [*n* (%)]	250 (47.35)	96 (42.29)	0.201
Total cholesterol within normal ranges [*n* (%)]	255 (48.30)	103 (45.37)	0.461

SD standard deviation

Table S3 The implementation details of the comparison methods

Methods	Architecture details	Learning rate tuned range	Batch size	Maximum number of epochs	Optimizer	Loss function
mcVAE	Multichannel variational autoencoder designed for joint latent and a ResNet50-based deep regressor	[0.0001, 0.0002]	16	1000	Adam optimizer	Reconstruction loss and KL divergence loss
Pixel2Pixel	A conditional generative adversarial network (cGAN) designed for image-to-image translation tasks	[0.0001, 0.0002]	16	1000	Adam optimizer	Adversarial loss and L1 loss
TransUNet	Transformer-based encoder and a U-Net-based decoder	[0.0001, 0.0002]	16	1000	Adam optimizer	Dice loss and cross-entropy loss
MT-Net	Multi-scale transformer encoder and a U-Net-based decoder	[0.0001, 0.0002]	16	1000	Adam optimizer	L1 loss and perceptual loss
ResViT	Residual connections with a vision transformer	[0.0001, 0.0002]	16	1000	Adam optimizer	L1 loss and adversarial loss

mcVAE multi-channel Variational Autoencoder, *Pixel2pixel* Pixel-to-Pixel, *TransUNet* transformer-based U-Net, *MT-Net* multi-scale transformer network, *ResViT* residual vision transformer, *CMCR* cross-modal correlation representation, *KL* Kullback-Leibler

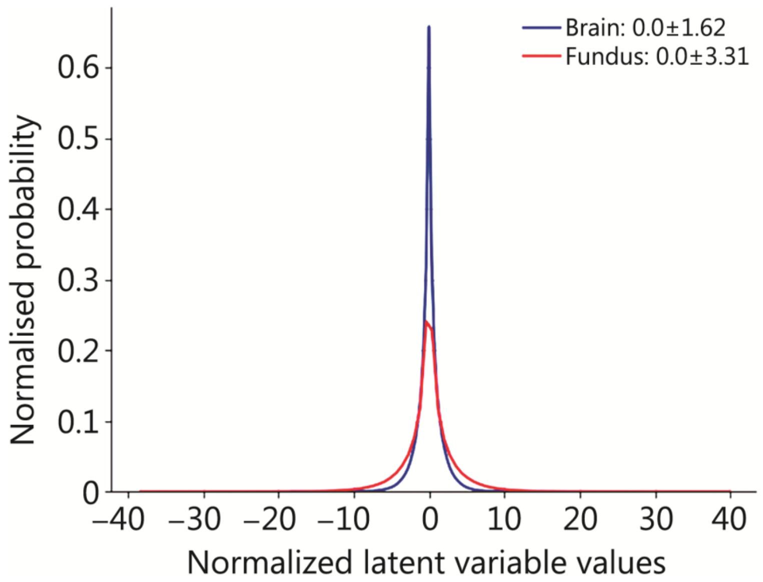

Fig. S1 Comparison of the latent space variance: distribution of the latent variables obtained using the proposed model on retinal fundus and brain images. The horizontal axis represents the normalized latent variable values, while the vertical axis denotes their probability density. The blue and red curves illustrate the distributions of latent variables derived from brain magnetic resonance imaging (MRI) and retinal fundus images, respectively. The numerical values in the legend represent the mean and standard deviation of latent variable distributions derived from the encoded representations of brain and retinal fundus images

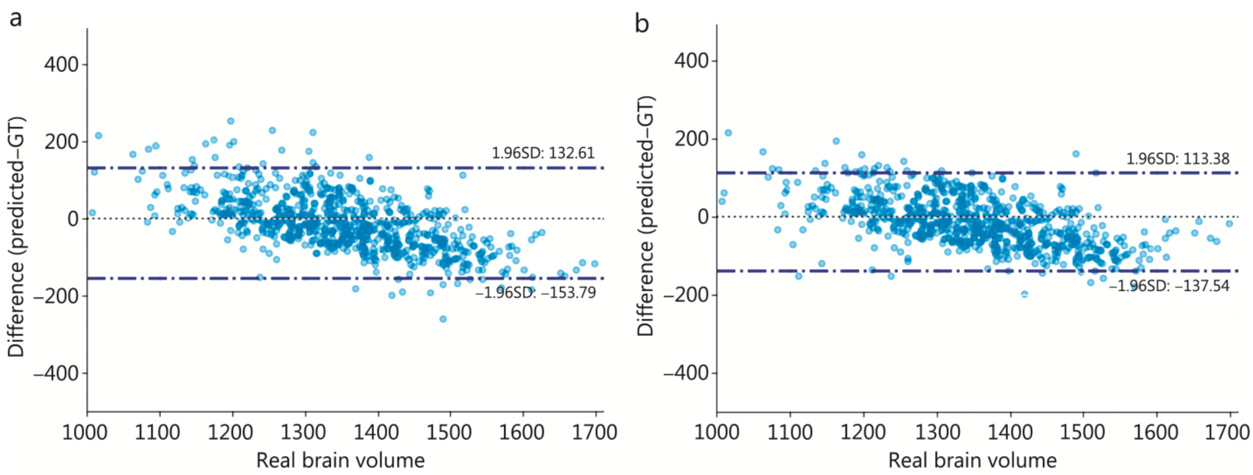

Fig. S2 Bland-Altman plots for estimated brain volumes and ground truths, without (**a**) and with (**b**) the incorporation of clinical information. GT ground truth

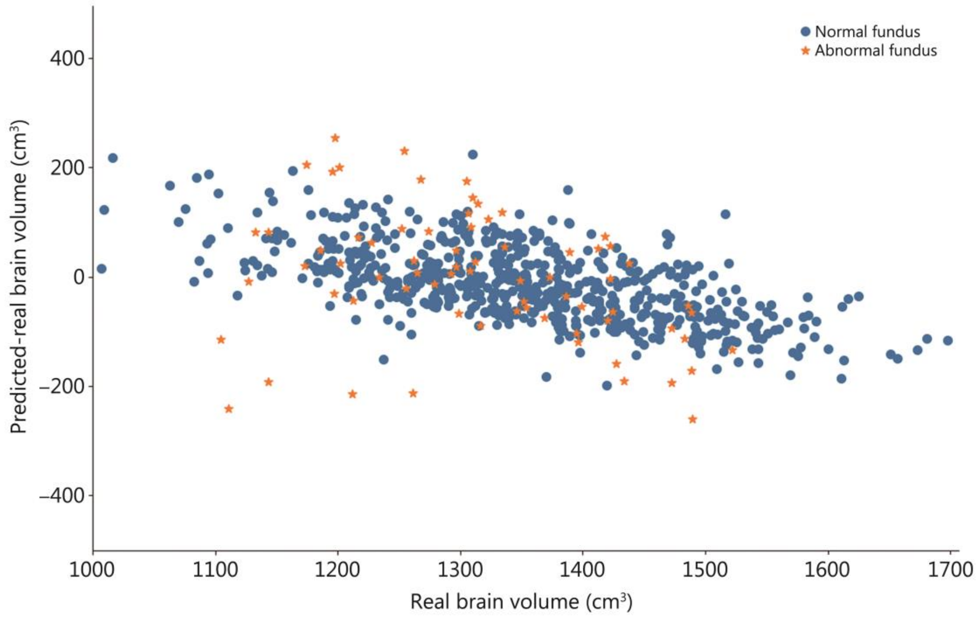

Fig. S3 Scatter plots of the estimated brain volume and the actual brain volume for subjects with normal and abnormal fundus conditions

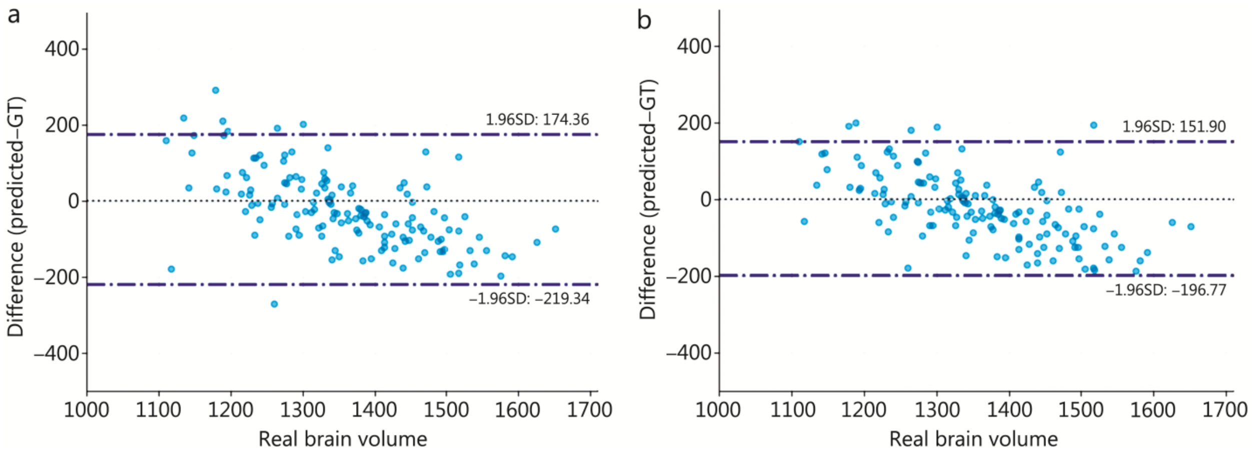

Fig. S4 Bland-Altman plots for estimated brain volumes and ground truths, without (**a**) and with (**b**) the incorporation of clinical information on the external validation set. GT ground truth
